# Global transcriptome profile reveals abundance of DNA damage response and repair genes in individuals from high level natural radiation areas of Kerala coast

**DOI:** 10.1371/journal.pone.0187274

**Published:** 2017-11-21

**Authors:** Vinay Jain, Birajalaxmi Das

**Affiliations:** 1 Low Level Radiation Research Section, Radiation Biology and Health Sciences Division, Bio-Science Group, Bhabha Atomic Research Centre, Trombay, Mumbai, India; 2 Homi Bhabha National Institute, Anushakti Nagar, Mumbai, India; Texas Tech University, UNITED STATES

## Abstract

The high level natural radiation areas (HLNRA) of Kerala coast in south west India is unique for its wide variation in the background radiation dose (<1.0mGy to 45mGy/year) and vast population size. Several biological studies conducted in this area did not reveal any adverse effects of chronic low dose and low dose rate radiation on human population. In the present study, global transcriptome analysis was carried out in peripheral blood mono-nuclear cells of 36 individuals belonging to different background dose groups [NLNRA, (Group I, ≤1.50 mGy/year) and three groups of HLNRA; Group II, 1.51–5.0 mGy/year), Group III, 5.01-15mGy/year and Group IV, >15.0 mGy/year] to find out differentially expressed genes and their biological significance in response to chronic low dose radiation exposure. Our results revealed a dose dependent increase in the number of differentially expressed genes with respect to different background dose levels. Gene ontology analysis revealed majority of these differentially expressed genes are involved in DNA damage response (DDR) signaling, DNA repair, cell cycle arrest, apoptosis, histone/chromatin modification and immune response. In the present study, 64 background dose responsive genes have been identified as possible chronic low dose radiation signatures. Validation of 30 differentially expressed genes was carried out using fluorescent based universal probe library. Abundance of DDR and DNA repair genes along with pathways such as MAPK, p53 and JNK in higher background dose groups (> 5.0mGy/year) indicated a possible threshold dose for DDR signaling and are plausible reason of observing *in vivo* radio-adaptive response and non-carcinogenesis in HLNRA population. To our knowledge, this is the first study on molecular effect of chronic low dose radiation exposure on human population from high background radiation areas at transcriptome level using high throughput approach. These findings have tremendous implications in understanding low dose radiation biology especially, the effect of low dose radiation exposure in humans.

## Introduction

Studying the biological effects of low dose and low dose-rate ionizing radiation (IR) in humans has important implications to human health and radiation protection science. Increasing evidences suggest that the effect of IR at low and high doses of exposures is qualitatively and quantitatively different [1,2]. At high acute dose exposures, IR may lead to deleterious effects at cellular and molecular level thus leading to adverse consequences like cell death, accumulation of mutations, chromosomal aberrations and carcinogenesis. However, there is still uncertainty regarding the risk/effect due to low dose and low dose rate exposures to humans, below 100 mSv. At such low dose exposures, biological mechanisms such as adaptive response, bystander effects, hypersensitivity and genomic instability may play important role which is not clearly understood yet. Hence, efforts have been made to undertake studies to strengthen future areas of research on biological effects of IR at cellular, molecular and /or single cell level [3].

The linear no threshold (LNT) hypothesis is based on the fact that even the smallest doses of radiation exposure have the potential to cause an increase in health risk to humans. However, the extrapolation of data from high dose exposures to low dose region of the dose response curve is one of the criticisms about LNT. Also, defence mechanisms such as DNA repair processes and elimination of damaged cells have not been taken into consideration for LNT hypothesis [4]. DNA repair process helps the cells to recover from various types of DNA lesions produced by a spectrum of genotoxic agents including IR. However, the threshold dose at which DNA damage response signal functions at chronic low dose exposures is not yet known.

In daily life, humans are exposed to low dose and low dose-rate ionizing radiation from various sources such as natural background, occupational, accidental, medical applications (diagnostic and therapeutic) etc. Natural chronic low dose exposure prevailing in high level natural radiation areas (HLNRA) in the world offers unique opportunity to study the biological effects of low dose radiation directly on humans. The HLNRA of Kerala is a narrow coastal line extending from Neendakara panchayat (Kollam district) in the south to Purakkad panchayat (Allapuzzha district) in the north. It has patchy distribution of monazite in the beach sand containing 8–10% of thorium-232 and therefore the human population (approximately 4,00, 000) is exposed continuously to natural chronic low dose radiation. Because of varied level of background radiation dose (<1.0 to 45.0 mGy/year), this area is suitable for dose response studies. Studying the biological effect of radiation exposure in humans at low dose and low dose-rates especially below 100mSv is highly challenging, but extremely essential for risk estimation. Human population residing in high background radiation areas provide the most appropriate opportunities to study the effect of IR at such low doses as they have been exposed continuously at all stages of development/organogenesis from birth to death.

Several investigations have been carried out in this area, which includes studies in wild rats[5], plants [6,7], demographic characterization of human population[8]. In addition, epidemiological studies are carried out to find out cancer incidence in adults, congenital anomalies in newborns and a case control study of mental retardation and cleft lip/palate in this population [9,10,11,12,13]. So far, no significant changes are observed at phenotype level. This population has also been investigated for several biological end points such as chromosome aberrations, micronuclei, telomere length measurement and quantitation of DNA damage [14,15,16,17,18,19,20,21,22].None of the above DNA damage end points have shown significant difference between the population from HLNRA and the adjacent normal level natural radiation areas (NLNRA). The spontaneous level of DNA double strand breaks (DSBs) has not shown any increase in DSBs, rather showed marginal reduction in HLNRA individuals belonging to background dose group >5mGy/year [18]. Interestingly, DNA damage and repair study in peripheral blood mono-nuclear cells (PBMC) using gamma H2AX marker and comet assay revealed that individuals from HLNRA groups (>5.0mGy/year) have better repair efficiency as compared to NLNRA group [21,23]. Our group has also shown *in vivo* radio-adaptive response among HLNRA group of individuals after a challenge dose of 1.0 and 2.0Gy of gamma radiation[24].

IR induces a spectrum of DNA damages in human cells that gets repaired through efficient DNA repair pathways depending upon the type of lesions. DSBs are highly deleterious. Other damages such as oxidized purines or pyrimidines, abasic sites, single strand breaks and clustered DNA lesions cannot be underestimated because un-repaired or mis-repaired DNA damages may lead to accumulation of mutations, chromosomal aberrations and carcinogenesis. DNA damage response (DDR) may signal a number of cellular and molecular pathways which alter the expression profile of many genes, proteins and regulating miRNAs, that may help in maintaining genome integrity. Studies have shown that human PBMC exposed to moderate doses of acute gamma radiation leads to alteration of expression profile of DDR and DNA repair genes and proteins [25,26,27,28,29].

There is a growing interest in the emerging areas of low dose radiation biology and development of biomarkers of radiation exposures. Development of new biomarkers may be useful for identifying radiation signatures in population exposed to radiation, accidental exposure situation like Chernobyl and Fukushima Daiichi nuclear disasters, biological dosimetry and population monitoring purposes [30,31,32,33,34,35,36]. There are established biological end points, which are used as radiation induced markers in the genome such as dicentrics, translocations and inversions. The sensitivity of these assays is low as compared to newly developed high throughput molecular biology techniques. In recent years, sensitive and specific assays such as gamma-H2AX foci, gene expression profile, miRNA and protein profile have emerged as biomarkers for radiation exposure [18,37,38,39,40].

Global gene expression profile in circulating lymphocytes provides rapid, non-invasive method to identify differential expression of genes in response to a variety of genotoxic agents including IR. There are very few reports available on acute and chronic low dose exposures to human population, where gene expression changes at specific genes are used as radiation signatures [30,32–33,35,36,41–45].

Global transcriptional profiling has been used to gain insights into the molecular mechanisms induced by low dose exposures in a variety of cell types and cell cultures such as human myeloid cells [1,46], human skin fibroblasts and keratinocytes [2,47,48], PBMC [41,44,49,50], umbilical vein endothelial cells [51], lymphoblastoid cells [52,53] and human embryonic stem cells [54]. There are reports, which have shown the induction of transcriptional changes after ex-vivo acute exposure to doses as low as 1 cGy [55]. Studies have also shown changes in transcriptional profile after *in vivo* low-dose exposures. For instance, Yin et al. (2003) identified several genes with modulated transcript levels after exposures of 10cGy in brain tissue from irradiated mice. However, limited information is available, where *in vivo* gene expression profile is carried out in human PBMC exposed to chronic low dose radiation such as occupational and accidental exposures [41,49,50]. Most importantly, till date, no report is available on gene expression profile in PBMC of human population living in high level natural radiation areas. It is also important to find out the threshold dose at which differential gene expression at low doses can be seen in humans. In the present study, attempt has been made to study global gene expression profile in PBMC of individuals from normal and high level natural radiation areas to find out differentially expressed genes and their role in various cellular and molecular pathways in response to chronic low dose and/or low dose rate radiation exposure.

## Materials and methods

### Ethics statement, study design and sample collection

Blood samples were collected from 36 healthy male individuals (volunteers) with written informed consent, which was approved by Medical Ethics Committee, Bhabha Atomic Research Centre, Trombay, Mumbai, India. All the methods were carried out in "accordance" with the approved guidelines by the above committee. Approximately, 10 ml of blood samples were collected in sterile EDTA containing vaccutainers (BD vaccutainers systems, U.S.A) from each individual (age range: 28–52 year). Individuals were stratified into four different dose groups based on the annual background dose received by them i.e., Group I (NLNRA, control group ≤ 1.5 mGy/year, N = 9), Group II (HLNRA, 1.51–5.0 mGy/year, N = 9), Group III (HLNRA, 5.01–15.0 mGy/year, N = 11) and Group IV (HLNRA, > 15.0 mGy/year, N = 7). All these individuals studied from different dose groups were having similar life style and dietary habits without any chronic illness. The information on age, gender, personal habits such as smoking, chewing, occupation, previous radiation exposure and medical history was collected in a detailed questionnaire. The overall mean age of 36 individuals was 40.4 ±5.7 years (age range: 28–52 years), which was similar to mean age of individuals in different background dose groups. The mean age among the individuals from Group I, Group II, Group III and Group IV was 39.7±4.7, 41.9±6.8, 40.5±6.9 and 39.3±4.3 years, respectively.

### Individual dosimetry

The external gamma-radiation levels were measured using a halogen quenched Geiger Muller (GM) tube-based survey meter (Type ER-709, Nucleonix Systems, India) in each individual’s house. The survey meter readings measured absorbed doses in air (μR h^-1^) due to gamma rays and were converted to annual dose (mGy year^-1^) using a conversion factor of 0.0765 (= 0.873 ×24 h × 365 days ×10^−5^). The individual dose contributed by the gamma rays was derived as sum of 0.5 (occupancy factor) × the annual indoor dose and 0.5 (occupancy factor) × the annual outdoor dose. The occupancy factor taken for the calculation of individual dose was based on the sex and age specific occupancy factors estimated in a previous study conducted by Nair et al, 2005 [56].

### Isolation of peripheral blood mononuclear cells (PBMC)

PBMC were isolated by density gradient centrifugation using Histopaque-1077 solution (Sigma-aldrich, St. Louis, MO, USA). Equal volume of blood was layered over histopaque solution and centrifuged at 400g for 30 minutes at room temperature. After centrifugation, opaque interface layer containing mono-nuclear cells was carefully separated and washed with chilled isotonic phosphate buffered saline (PBS) and centrifuged at 250g for 10 minutes. The cell pellet was washed twice with PBS. The cells were divided into small aliquots, suspended in RNA later (Sigma-aldrich) and stored at—20°C until further use.

### Extraction of total RNA

Total RNA was isolated from PBMC using RNeasy Mini Kit (Qiagen, Valencia, CA, USA) as per manufacturer’s instructions. Quality and quantity of isolated total RNA was checked using Nano Drop^®^ ND-1000 spectrophotometer (Nano Drop Technologies, USA). The integrity of isolated RNA was evaluated with microfluidic capillary electrophoresis using the Agilant 2100 Bioanalyzer (Agilant technologies, U.S.A). RNA integrity number (RIN) was calculated for all the extracted RNA samples. RIN values ≥ 8.0 were taken for microarray experiment as well as validation of genes in real time quantitative PCR (RT q-PCR).

### Preparation of cRNA and hybridization using Affymetrix Gene Chip

Biotin labelled cRNA was prepared using Gene Chip^®^3′ IVT Express Labeling Kit (Affymetrix, Santa Clara, CA, USA). All the procedures followed were as per the manufacturer’s protocol (Affymetrix). Briefly, 100 ng of total RNA was used to synthesize double stranded cDNA using an oligo(dT) primer containing the T7 RNA polymerase promoter site provided with GeneChip^®^3′ IVT Express Labeling Kit (Affymetrix, Santa Clara, CA). Purification of cRNA was done to remove unincorporated nucleotide triphosphates, salts, enzymes, and inorganic phosphates prior to fragmentation and hybridization onto Human Genome U133 Plus 2.0 Gene Chip expression arrays. Genome arrays were hybridized for 16 hours overnight at 45°C as per the affymetrix protocol. Following washing and staining, the Gene Chips were scanned using the Affymetrix Gene Chip Scanner 3000. The raw data file formats were generated using Gene Chip operating software (GCOS).

### Differential gene expression and statistical analysis

All the samples were normalized, filtered and analysed using R software. Robust Multichip Analysis (RMA) normalization method was used for background correction, normalization and calculation of expression values. COMBAT software developed by Johnson et al (2007) [57] was used to adjust the expression values of samples processed in different batches. The normalized expression data was used to identify probe sets/genes that are differentially regulated across the comparison of interest. Identification of differentially expressed genes was performed with the Linear Models for Microarray Data (LIMMA) package available on Bio-Conductor. To obtain differentially expressed genes, moderated t-statistic was applied. Multiple testing corrections were performed using Benjamin & Hochberg (BH) correction adjusting for the false discovery rate (FDR). A threshold adjusted p-value was set < 0.05, and the fold-change threshold was set to 1.3. These settings were retained throughout the analysis to select gene list across different group comparisons.

### Gene ontology and pathway analysis

The list of genes obtained through various comparisons was studied for their overabundance in different GO terms as well as Pathways. Fisher’s exact test was used to determine the significance of the GO analysis and significance level p < 0.05 was set for enriched genes and biological relevance of the associated genes was explored. Bioinformatic analysis was carried out using Genowiz^™^ ver. 4.0 (Ocimum Biosolutions, Hyderabad, India) software. In the present study, for GO analysis, the data from Gene Ontology consortium was used, while for pathways, human KEGG pathways were referred.

### Validation of microarray data using real time quantitative PCR (RT-qPCR)

The differentially expressed genes from microarray results were selected for validation using fluorescent based hydrolysis probes in Light Cycler 480 (Roche Diagnostics Pvt. Ltd, GmbH, Germany). Two independent groups of samples were taken for validating the microarray data. The first group consisted of 30 individuals which were included in microarray study. Validation was not possible for six individuals due to the limitation of samples in that particular aliquot (cDNA prepared for these six samples were insufficient to carry out validation in real time q-PCR). The second group consisted of freshly collected independent set of 24 individuals from Kerala coast. For each sample, total RNA was isolated, checked for purity and integrity. cDNA was synthesized using High fidelity cDNA synthesis Kit (Roche Diagnostics). For all the genes, intron spanning PCR primers were designed using Probe finder software version 1.1 and specific hydrolysis probes from Universal Probe library set for Humans (Roche Diagnostics) were used. Primer sequences used in the study are given in S1 Table. RT-q PCR reactions were performed on 96 multi-well plates in triplicates and ß-actin (ACTB) was used as a housekeeping gene for normalization. Fold change was calculated by dividing average normalized value in HLNRA individuals with average normalized value in NLNRA individuals.

## Results

**Fig 1:**
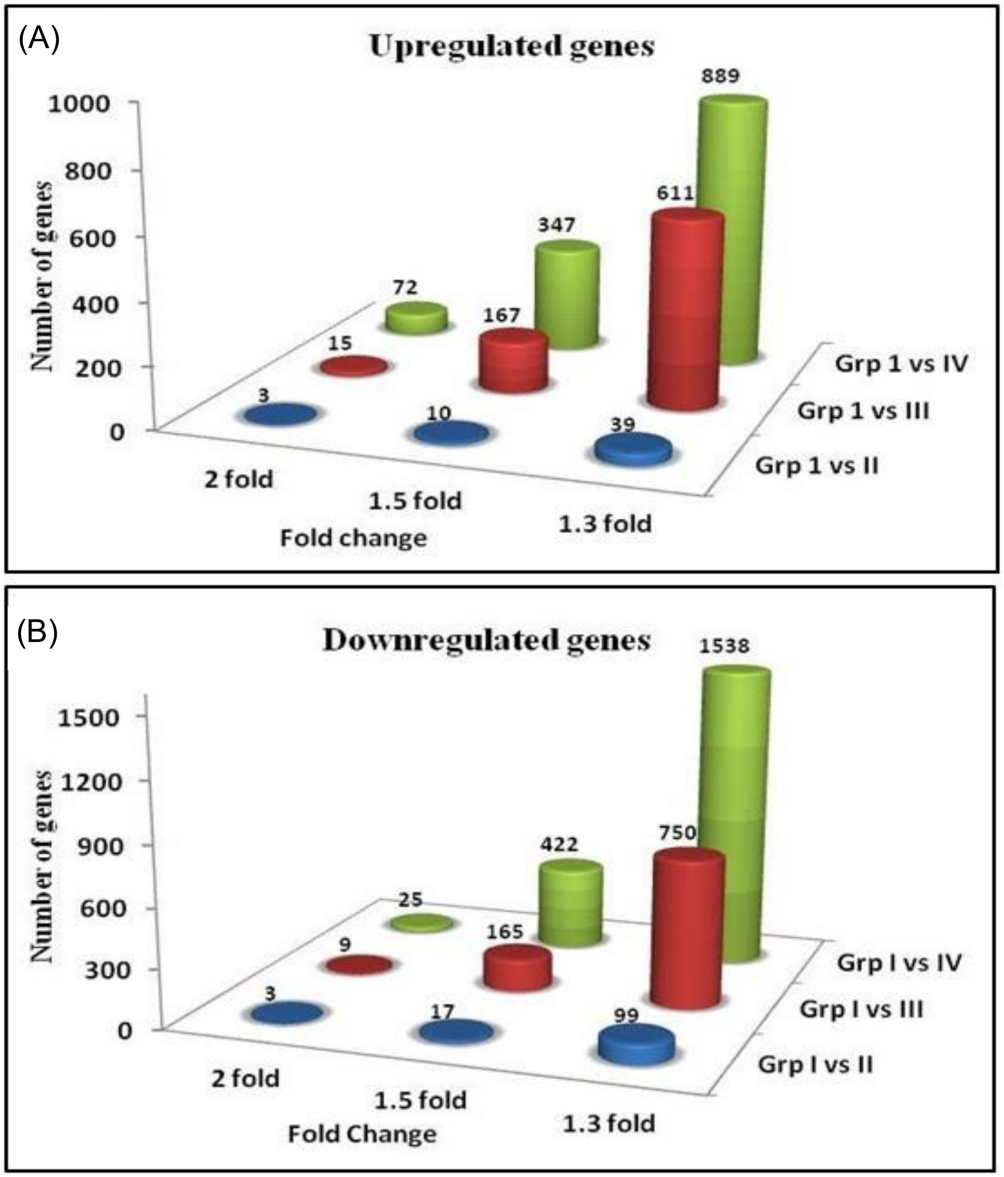
Histograms showing the number of differentially expressed genes in different HLNRA groups Grp II, Grp III and Grp IV) with respect to NLNRA group (Grp I). (a) Upregulated genes with adjusted p value < 0.05 (b) Downregulated genes with adjusted p value < 0.05.

Transcriptome analysis was carried out in PBMC of 36 random, healthy individuals belonging to different background dose groups [Group I (NLNRA; ≤ 1.5 mGy/year, N = 9), Group II (HLNRA; 1.51–5.0mGy/year, N = 9), Group III (HLNRA; 5.01–15.0 mGy/year, N = 11), and Group IV (HLNRA; > 15.0 mGy/year, N = 7)] from Kerala coast using affymetrix Human Genome U133 Plus 2.0 Gene Chip. The objective was to find out differentially expressed genes in different HLNRA dose groups (Group II, Group III and Group IV) with respect to NLNRA (control group) and their biological significance in response to chronic low dose radiation. The NLNRA (group I) group of individuals were considered as control population and the expression profile observed in this group was considered as background level changes in gene expression at a particular gene. Analysis was carried out to identify differentially expressed genes at a threshold fold change value of 1.3, 1.5 and 2.0 fold with adjusted p-value < 0.05. The differentially expressed genes (up and down-regulated) in each HLNRA dose group (Group II, Group III and Group IV) as compared to NLNRA (Group I) is given in Table 1. At a fold change threshold value of 2.0, only 6 genes (3 Up and 3 down) were differentially expressed in Group I vs. II, 24 (15 up and 9 down) genes in Group I vs. III and 97 genes (72 up and 25 down) in Group I vs. IV, respectively.At a fold change threshold value of 1.5, we observed 27 (10 up and 17 down) genes were differentially expressed in Group I vs. II, 332 genes (167 up and 165 down) in Group I vs. III and 769 genes (347 up and 422 down) in Group I vs. IV, respectively (Fig 1).

**Table 1:** Number of differentially expressed genes (up and down-regulated) in different HLNRA dose groups with respect to NLNRA (control group) at different fold changes (1.3, 1.5 and 2.0) with p < 0.05. Group I refers to background dose level ≤ 1.5mGy/y, Group II: 1.51–5.0 mGy/y, Group III: 5.01–15.0 mGy/y and Group IV: >15.0 mGy/y.

Background Dose Groups	P-value set at < 0.05					
	Fold change 1.3		Fold change 1.5		Fold change 2.0	
	Up	Down	Up	Down	Up	Down
NLNRA (Group I) vs HLNRA (Group II) (≤ 1.5mGy/y vs 1.51–5.0 mGy/y)	39	99	10	17	3	3
NLNRA (Group I) vs HLNRA (Group III) (≤ 1.5mGy/y vs 5.01–15.0 mGy/y	611	750	167	165	15	9
NLNRA (Group I) vs HLNRA (Group IV) (≤ 1.5mGy/y vs >15.0 mGy/y)	889	1538	347	422	72	25

**Fig 2:**
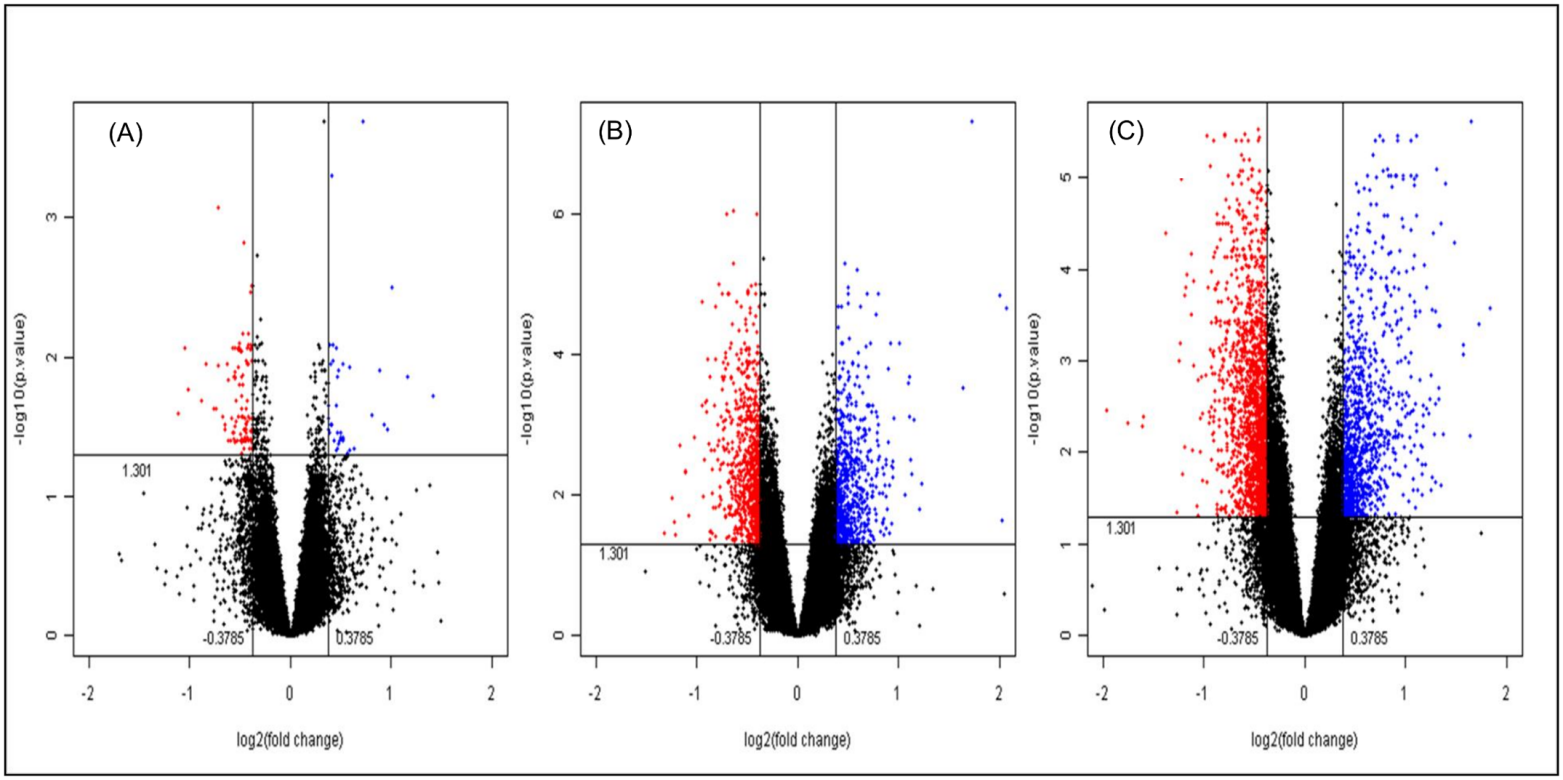
Volcano plots representing the differentially expressed genes (up and down-regulated) at 1.3 fold in different HLNRA groups (Group II, III and IV) as compared to NLNRA (Group I). A) Group I vs. II, B) Group I vs. III, C) Group I vs. IV. Blue dots represent significant (p<0.05) up-regulated genes and red dots represent significant down- regulated genes.

In order to get a large number of biologically significant genes from various molecular and cellular pathways that are differentially expressed in different background dose groups, analysis was carried out at a fold change threshold of 1.3 and adjusted p-values < 0.05. Our data revealed a total of 138 (39 up and 99 down), 1361 (611 up and 750 down) and 2427 (889 up and 1538 down) genes to be differentially expressed between Group I vs. Group II, Group I vs. Group III and Group I vs. Group IV, respectively (Figs 1 and 2). Interestingly, we observed a background dose related increase in the number of differentially expressed genes in different HLNRA groups as compared to NLNRA group.

### Identification of common genes among different background radiation dose groups

**Fig 3:**
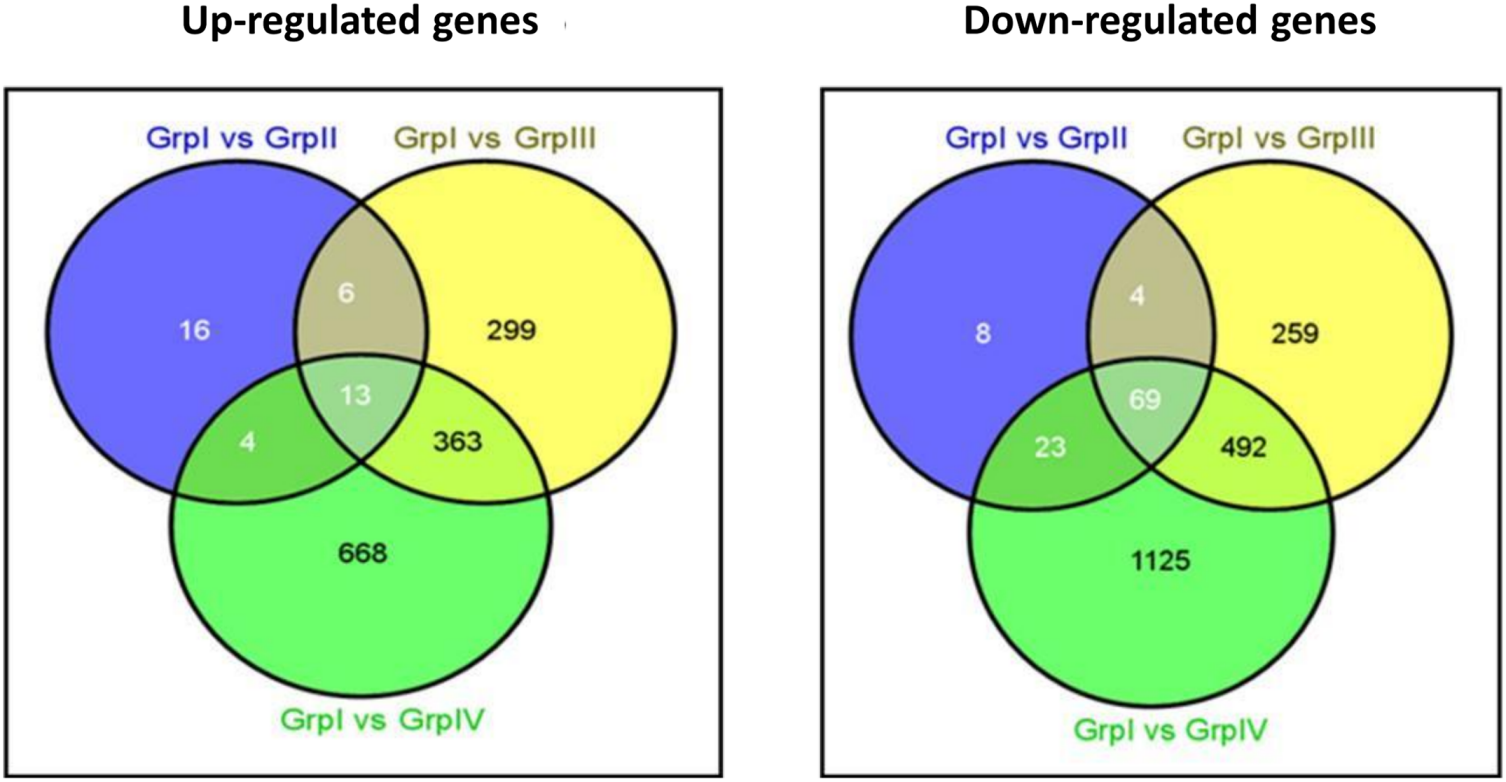
Venn diagram showing common as well as unique genes expressed in different group comparisons. Genes showing ≥ 1.3 fold change was considered for analysis.

As shown in the Venn diagram (Fig 3), a total of 82 common genes (13 up-regulated and 69 down-regulated) were found to be differentially expressed in all the three HLNRA groups. Further analysis showed a total of 92 (19 up and 73 down), 109 (17 up and 92 down) and 937 (376 and 561) genes were common between Group II and III, Group II and IV and Group III and IV, respectively. Similarly, a total of 24 (16 up and 8 down), 558 (299 up and 259 down) and 1793 (668 up and 1125 down) genes were uniquely expressed in Groups II, III and IV, respectively. Interestingly, higher number of common genes was observed in high dose groups (Group III and Group IV). Detailed characteristics and function of the common genes are given in Tables 2 and 3. We have observed some of the important up-regulated (*EIF1*, *PDE4B*, *USP36* and *SNRPA1)* and down-regulated (*NT5E*, *NUPL2*, *GTF2E1*, *GRB10*, *IL16*, *SLC4A7*, *METTL13*, *TMEM184C*, *TRIM36*, *PPIL1*, *KDM5A*, *NDUFAF4*, *TUBD1*, *DHFRL1)* common genes among the three groups. These common genes are involved in RNA processing, cell cycle regulation, apoptosis, microtubule formation, nucleotide metabolism, transcription initiation and regulation, cell growth, signal transduction, protein folding, cytokine activity and ion transport etc.

**Table 2:** Set of differentially expressed genes showing up-regulation at > 1.3 fold change in three HLNRA dose groups (Grp II, Grp III, Grp IV) with respect to NLNRA (Grp I) with adjusted p- value < 0.05.

Gene Symbol	Accession number	Fold change Grp I vs Grp II	Fold change Grp I vs Grp III	Fold change Grp I vs Grp IV	Function
ZBTB24	BC036731	1.38	1.39	1.45	Transcription regulation
EIF4G3	BC030578	1.55	1.59	1.87	Translation initiation
PDE4B	L20966	1.85	1.66	2.04	Signal transduction
EIF1	AL516854	1.50	1.72	1.87	Translation initiation
HNRNPM	AK024911	1.42	1.39	1.41	RNA processing
AF070620	AF070620	1.64	1.61	1.63	-
USP36	NM_025090	1.75	1.54	2.27	Protein degradation
RPL10	AW057781	1.39	1.38	1.41	Ribosome synthesis
USP15	AK023703	1.44	1.32	1.52	Protein degradation
AK024583	AK024583	2.01	3.30	3.14	-
AI684439	AI684439	2.67	4.19	3.56	-
AW009425	AW009425	1.39	1.44	1.37	-
SNRPA1	AA872471	1.47	1.67	1.41	RNA splicing

**Table 3:** Set of differentially expressed genes showing down-regulation at > 1.3 fold change in three HLNRA dose groups (Grp II, Grp III, Grp IV) with respect to NLNRA (Grp I) with adjusted p- value < 0.05.

Gene Symbol	Accession number	Fold change Grp I vs Grp II	Fold change Grp I vs Grp III	Fold change Grp I vs Grp IV	Function
NT5E	BC015940	-1.69	-1.60	-1.81	Nucleotide metabolism
LOC100287896	BC019340	-1.64	-1.69	-1.97	**-**
BET1	BC000899	-1.50	-1.60	-1.61	Protein transport
NUPL2	NM_007342	-1.35	-1.38	-1.43	mRNA transport
ZBTB1	NM_014950	-1.48	-1.63	-1.67	Transcription regulation
GTF2E1	NM_005513	-1.44	-1.66	-1.82	Transcription initiation
ZNF167	NM_018651	-1.49	-1.68	-1.81	Transcription regulation
JRKL	NM_003772	-1.31	-1.43	-1.73	-
GRB10	D86962	-1.35	-1.31	-1.60	Insulin receptor binding
IL16	M90391	-1.54	-1.67	-1.56	Cytokine activity
SLC4A7	AF047033	-1.39	-1.48	-1.75	Ion transport
HGF	X16323	-1.48	-1.32	-1.55	Cell growth
FEM1B	AI799061	-1.33	-1.58	-1.66	Apoptosis
METTL13	AK001172	-1.41	-1.42	-1.62	Methyltransferase activity
MED20	AK023092	-1.31	-1.42	-1.35	Transcription regulation
KLHL9	AW138594	-1.40	-1.39	-1.42	Cell cycle regulation
TTC9	AW235608	-1.43	-1.31	-1.54	Cell growth
ISOC1	NM_016048	-1.36	-1.42	-1.33	-
SAR1B	NM_016103	-1.32	-1.44	-1.34	Protein transport
MANEA	NM_024641	-1.46	-1.43	-1.34	Protein transport
ETNK1	NM_018638	-1.31	-1.39	-1.32	Cell metabolism
TMEM184C	NM_018241	-1.31	-1.41	-1.44	Cell growth
BOLA1	NM_016074	-1.43	-1.46	-1.69	-
BBS10	NM_024685	-1.45	-1.65	-1.76	Protein folding
TRIM36	NM_018700	-1.40	-1.39	-1.35	Cell cycle regulation
L1TD1	NM_019079	-1.36	-1.50	-1.43	**-**
CALHM2	BC000039	-1.49	-1.37	-1.38	Ion transport
CCDC28B	AL049795	-1.36	-1.50	-1.35	Cilia development
PPIL1	BC003048	-1.42	-1.77	-1.63	Protein folding
TRNT1	BE552215	-1.54	-1.60	-1.59	Nucleotide metabolism
ARRB1	BC003636	-1.35	-1.45	-1.34	Signal transduction
GBP3	AL136680	-1.48	-1.42	-1.56	GTPase activity
EXOSC3	AF281132	-1.33	-1.47	-1.52	RNA processing
THAP2	AL136607	-1.66	-1.66	-1.90	Apoptosis
ANUBL1	AF311324	-1.36	-1.39	-1.39	Cell cycle regulation
YIPF5	AW001618	-1.64	-1.65	-1.86	Protein transport
SLC35F5	AA044835	-1.37	-1.39	-1.38	Ion transport
NAPEPLD	BF382393	-1.78	-1.52	-1.79	Cell metabolism
APH1B	AW237258	-1.38	-1.39	-1.44	Peptidase activity
KDM5A	AI672662	-1.39	-1.44	-1.65	Histone demethylation
TMTC3	AA428286	-1.35	-1.35	-1.50	Transmembrane protein
MED11	AL531790	-1.32	-1.55	-1.52	Transcription regulation
NDUFAF4	AL521129	-2.02	-2.03	-2.16	Mitochondrial respiratory chain
EXOSC3	AA747303	-1.40	-1.48	-1.50	RNA processing
ZNF322A	AW511258	-1.47	-1.31	-1.47	Transcription regulation
CCDC126	AK026684	-1.40	-1.57	-1.59	Cell metabolism
MFSD8	AW611550	-1.40	-1.38	-1.65	Ion transport
ZEB2	AI912571	-1.55	-1.66	-1.70	Transcription regulation
W46994	W46994	-1.34	-1.38	-1.52	-
PPM1N	BE732320	-1.38	-1.37	-1.39	Cell cycle regulation
ZNF823	AI417785	-1.46	-1.47	-1.57	Transcription regulation
ZNF585A	BE550717	-1.42	-1.59	-1.48	Transcription regulation
KCTD21	AI391633	-1.37	-1.60	-1.74	Ion transport
LPAR5	AW183080	-1.47	-1.64	-1.66	Signal transduction
AI673025	AI673025	-1.41	-1.35	-1.35	-
TUBD1	AK022771	-1.43	-1.62	-1.70	Microtubule formation
BPNT1	AI439695	-1.35	-1.45	-1.73	Nucleotide metabolism
ARL6	AL138043	-1.37	-1.33	-1.65	Protein transport
DHFRL1	AW104373	-1.51	-1.65	-1.82	Cell metabolism
AI823600	AI823600	-1.59	-1.49	-1.76	-
AI738675	AI738675	-1.40	-1.58	-1.47	-
ZNF738	AI758317	-1.57	-1.49	-1.48	Transcription regulation
AW770320	AW770320	-1.31	-1.36	-1.59	-
R63578	R63578	-1.38	-1.48	-1.72	-
ZNF234	BE909177	-1.33	-1.34	-1.49	Transcription regulation
SOAT1	AA946876	-1.33	-1.56	-1.52	Cell metabolism
ABHD6	AA209239	-1.39	-1.61	-1.34	Cell metabolism
PIGV	AA203365	-1.38	-1.64	-1.47	Signal transduction

### Gene ontology analysis of differentially expressed genes in HLNRA groups as compared to NLNRA

**Fig 4:**
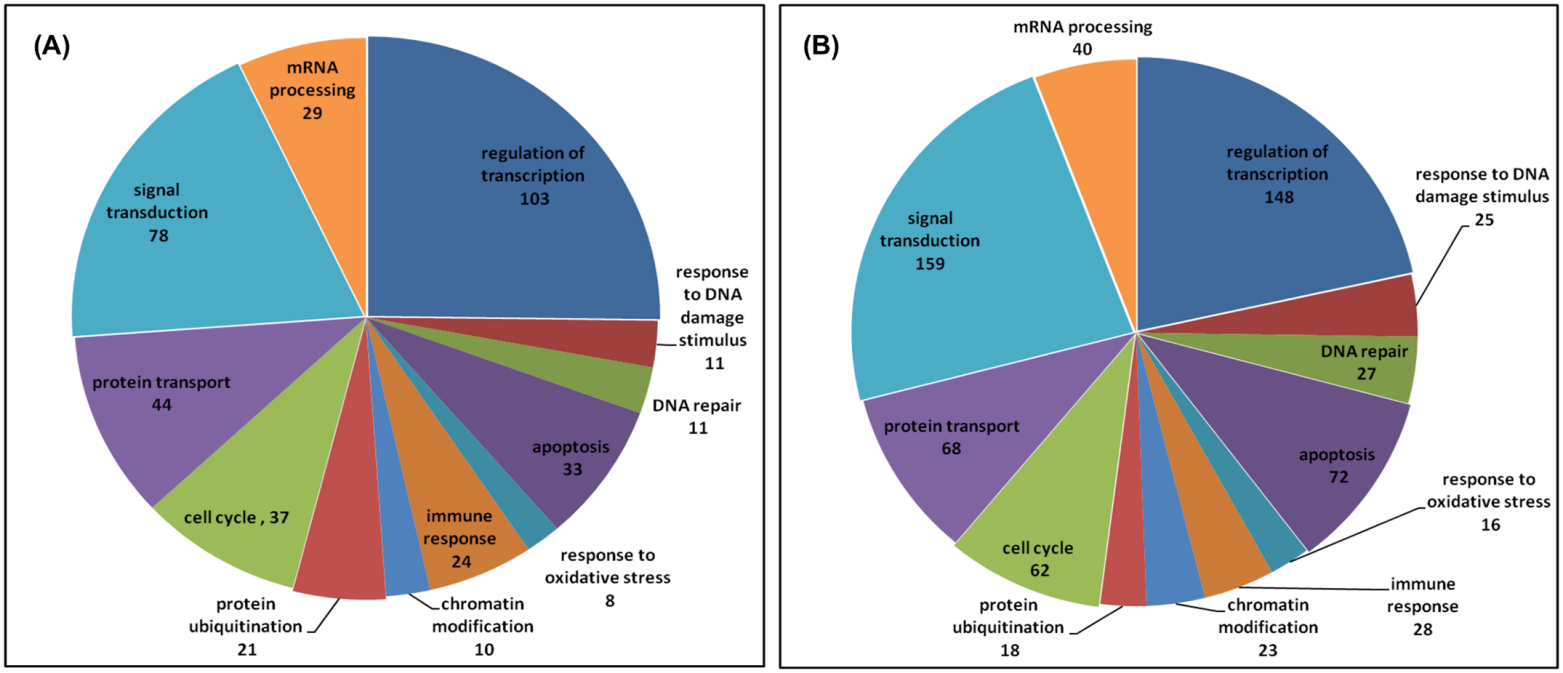
Representative biological processes (GO analysis) over-represented in differentially expressed genes of high dose groups A) Group III (5.01–15.0 mGy/y) and B) Group IV (>15.0 mGy/y) of HLNRA as compared to Group I (NLNRA).

Gene ontology analysis was carried out in order to find out the biological significance and molecular function of the differentially expressed genes. Analysis was done to find out over-represented biological processes, cellular components, molecular functions and critical pathways with a threshold fold change of 1.3 and a statistical significance of p <0.05. Overall analysis has revealed that biological processes such as regulation of transcription (*JUN*, *NR4A2*), apoptosis (*SIAH1*, *PMAIP1*), regulation of cell cycle (*CDKN1A*, *BTG1*), response to DNA damage (*GADD45B*, *DDIT3*), metabolic processes (*NAMPT*, *NT5E*), RNA processing (*PAPD4*, *SRRM1*), Immune response (*IL8*, *KIR3DS1*), signal transduction (*MAPK6*, *AKT2*), DNA repair (*POLB*, *LIG4*), protein transport (*SEC31A*, *NUPL1*), histone/chromatin modification (*SETDB2*, *SIRT2*), response to oxidative stress (*SOD2*, *OXR1*), protein ubiquitination (*USP36*, *UBE2B*.) are over-represented in higher dose groups (Group III and Group IV) as compared to Group I (Fig 4). In terms of cellular location, 23–29% genes coded for nuclear proteins, 15–22% coded for cytoplasmic proteins, 4–8% coded for mitochondrial genes and 15–17% were involved in membrane bound activities. Some of the significantly overrepresented molecular function in higher dose groups (Group III and IV) included transcription factor activity, protein kinase activity, DNA/RNA and protein binding, signal transducer activity, histone binding activity, metal ion binding, ligase activity, helicase activity, methyltransferase activity, ubiquitin-protein ligase activity and oxidoreductase activity.

### Pathway analysis of differentially expressed genes in HLNRA groups as compared to NLNRA

**Fig 5:**
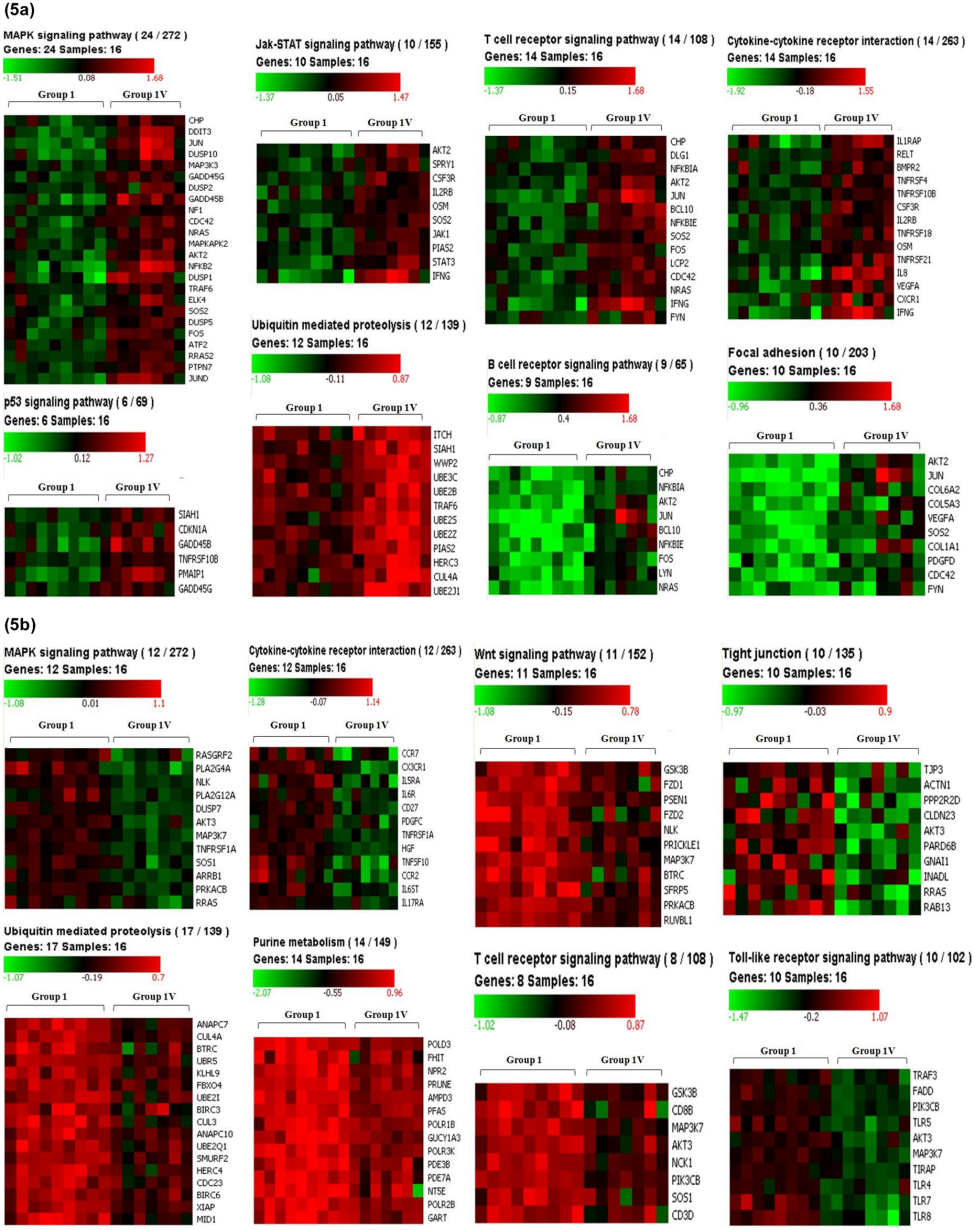
Representative heat maps for the respective pathways of up-regulated and down regulated genes in Group IV (high dose group, HLNRA) as compared to NLNRA (Group I). (a) Representative heat maps for up-regulated genes in Group IV vs Group I (b) Representative heap maps for down regulated genes in Group IV vs Group I. Color panel shows expression intensity levels of genes involved in each pathway.

Pathway analysis was done in all the groups (Group II, Group III and Group IV) as compared to NLNRA to find out relevant cellular and molecular pathways activated in response to chronic low dose exposure in HLNRA individuals. Our analysis showed that in higher dose groups (Group III and IV), various important pathways such as MAPK signaling, Jak-STAT signaling, p53 signaling, T cell receptor signaling, B cell receptor signaling, insulin signaling, purine metabolism, apoptosis, cell cycle, DNA repair, ubiquitin mediated proteolysis, focal adhesion, gap junction etc. were found to be over-represented (Table 4). Representative heat maps showing the genes from each pathway in higher dose groups especially Group IV (HLNRA) as compared to Group I (NLNRA) is shown in Fig 5A and 5B.

**Table 4:** Representative list of over-represented pathways in higher dose group (Group IV) as compared to NLNRA (Group I).

Over-represented Pathways	No. of genes / database count	P-value
***Up-regulated***		
MAPK signaling pathway	(24 / 272)	9.87E-013
T cell receptor signaling pathway	(14 / 108)	5.81E-010
B cell receptor signaling pathway	(9 / 65)	4.21E-007
Cytokine-cytokine receptor interaction	(14 / 263)	1.46E-005
Jak-STAT signaling pathway	(10 / 155)	5.34E-005
Ubiquitin mediated proteolysis	(12 / 139)	5.83E-007
p53 signaling pathway	(6 / 69)	0.0003
Focal adhesion	(10 / 203)	0.0004
Gap junction	(7 / 96)	0.0003
***Down-regulated***		
Ubiquitin mediated proteolysis	(17 / 139)	2.08E-007
Purine metabolism	(14 / 149)	3.73E-005
Insulin signaling pathway	(13 / 138)	6.82E-005
Toll-like receptor signaling pathway	(10 / 102)	0.0003
Tight junction	(10 / 135)	0.002
Wnt signaling pathway	(11 / 152)	0.001
T cell receptor signaling pathway	(8 / 108)	0.006
MAPK signaling pathway	(12 / 272)	0.04
Cytokine-cytokine receptor interaction	(12 / 263)	0.04

### DNA damage response and repair genes in HLNRA individuals

**Fig 6:**
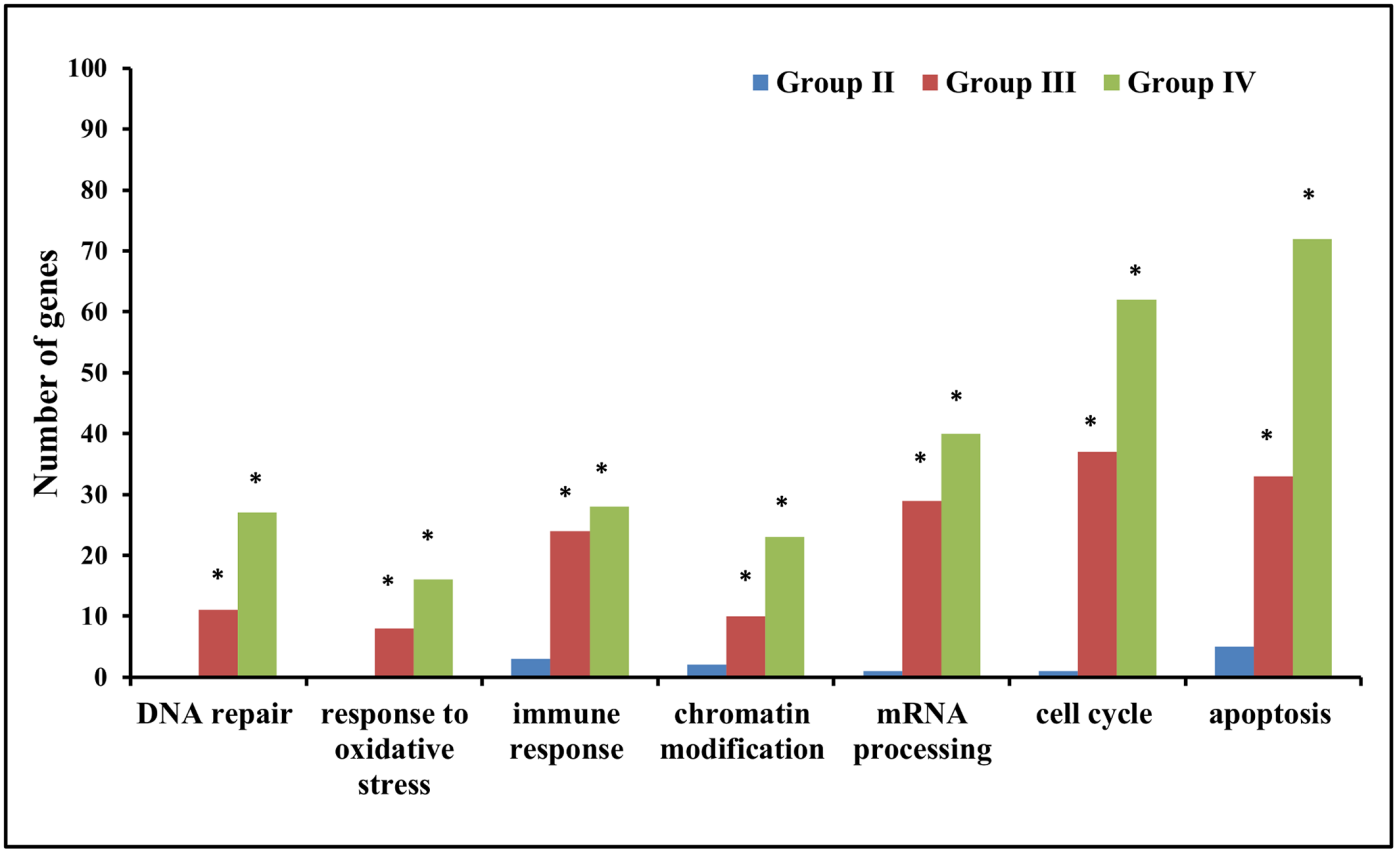
Gene ontology analysis showing over-representation of DDR related biological processes to be highly represented in high dose groups (Group III and Group IV) than in Group II. * shows statistical significance at p < 0.05.

Interestingly, our data revealed that differentially expressed genes in Group II are involved in biological processes such as immune response (*IL1A*, *IGHG1*), apoptosis (*WDR92*, *APH1B*), RNA splicing (*SNRPA1*, *HNRNPM*) and translational initiation (*EIF1*, *EIF4G3*) etc. However, high dose groups (>5.0 mGy/year) have higher representation/abundance of genes involved in DNA damage response that include DNA repair, response to oxidative stress, immune response, chromatin modification (methylation and histone modification), mRNA processing, cell cycle, and apoptosis (Fig 6).

**Fig 7:**
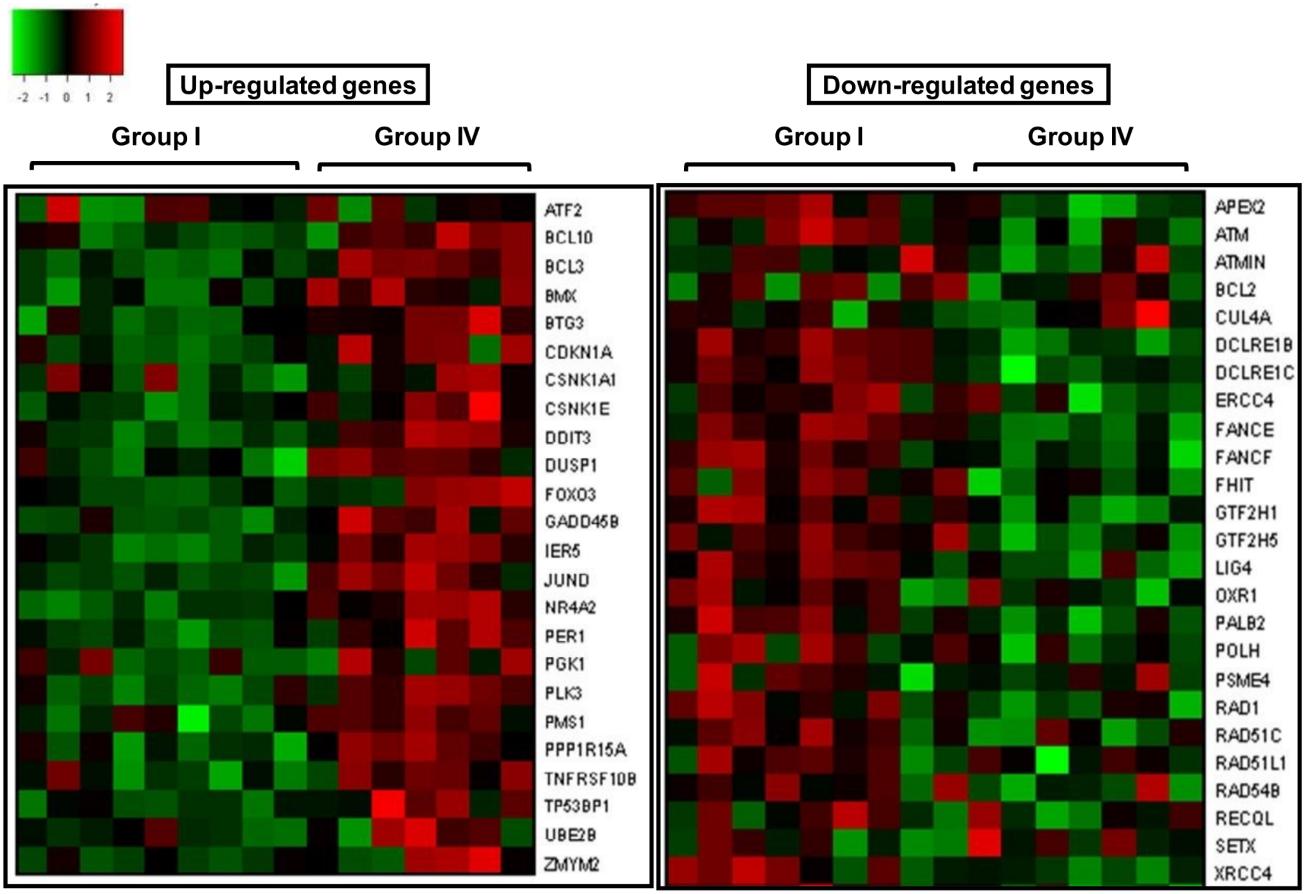
Representative heat maps showing intensity values of up and down regulated DNA damage response and repair genes in high dose group (Group IV) of HLNRA as compared to Group I.

Several genes involved in different DNA repair pathways were present in higher dose groups (Group III and Group IV: > 5.0 mGy/year). These includes genes involved in non-homologous end joining repair *(XRCC4*, *LIG4*, *DCLRE1C*, *DCLRE1B*, *DCLRE1A)*, base excision repair *(APEX2*, *UNG*, *POLB)*, nucleotide excision repair (*RAD23B*, *GTF2H1*, *GTF2H3*, *GTF2H5*, *ERCC4*), homologous recombination repair (*RAD54B*, *RAD51B*), mismatch repair (*PMS1)* and other repair genes (*RAD21*, *RAD 18*, *RMI1*, *POLH*, *UBE2B*, *UBE2T*, *FANCE*, *FANCF*, *PALB2*, *REV3L*, *HLTF*). DDR regulated pathways such as MAPK signaling, p53 signaling, JAK-STAT signaling were also activated in higher dose groups (Group III and IV). Although expression levels of p53 gene did not show any change, but p53 regulated genes such as *CDKN1A*, *TRAF4*, *ATF3*, *TNFRSF10B*, *APAF1*, *DUSP1*, *PMAIP1*, *GADD45B*, *BCL6*, *PLK3* etc were differentially expressed in our data. Some of the genes involved in MAPK pathway were *DUSP1*, *DUSP10*, *AKT2*, *ATF2*, *BCL10*, *CD38*, *CDC42*, *CSNK1A1*, *DDIT3*, *DUSP5*, *GFRAL*, *GNAS*, *HIPK3*, *IL8* etc. Several transcription factors and signaling molecules, which are involved in regulating DNA damage response and repair such as *cJUN*, *FOSB*, *JUND*, *CREBZF*, *FOXO3*, *ATF2*, *HEY1*, *NR4A2* etc were found to be activated in higher dose groups. A representative list of important genes involved in DDR processes is given in Table 5. Representative heat maps (up and down regulated genes) showing over-represented DDR and repair genes in higher dose group (Group IV) with respect to NLNRA (Group 1) is shown in Fig 7.

**Table 5:** List of important genes involved in various DNA damage response processes.

Gene name	Accession no.	Chromosomal location	Fold change Group II	Fold change Group III	Fold change Group IV
**A. DNA repair genes**					
*APEX2*	NM_014481	chr Xp11.21	-	-	-1.30
*UNG*	NM_080911	chr 12q24.11	-	-	-1.32
*XRCC4*	NM_003401	chr 5q14.2	-	-	-1.35
*LIG4*	NM_002312	chr 13q33.3	-	-1.32	-1.45
*DCLRE1C*	NM_022487	chr10 p13	-	-	-1.44
*DCLRE1B*	NM_022836	chr10p13.2	-	-	-1.35
*DCLRE1A*	NM_014881	chr10q25.1	-	1.41	-
*RAD51L1*	NM_002877	chr14q23	-	-	-1.32
*RAD54B*	NM_012415	chr8q21.3	-	-	-1.44
*RAD51C*	NM_002876	Chr17q22	-	-1.32	-1.36
*RAD21*	NM_006265	chr9q31.2	-	1.64	-
*RAD23B*	NM_002874	chr9q31.2	-	1.34	-
*RAD18*	NM_020165	chr3p25-p24	-	-1.31	-
*GTF2H1*	NM_001142307	chr11p15.1	-	-1.42	-1.45
*GTF2H5*	NM_207118	chr6q25.3	-	-	-1.31
*GTF2H3*	NM_001516	chr12q24.31	-	-	-1.46
*ERCC4 (XPF)*	NM_005236	chr16p13.12	-	-	-1.58
*PMS1*	NM_000534	chr2q31	-	-	1.57
*RMI1*	NM_024945	chr9q21.32	-	-1.50	-1.72
*POLB*	NM_002690	chr8p11.2	-	1.56	1.58
*POLH*	NM_006502	chr6p21.1	-	-1.33	-1.42
*UBE2B*	NM_003337	chr5q23-q31	-	1.34	1.36
*UBE2T*	NM_014176	chr1q32.1	-	-1.39	-1.47
*FANCE*	NM_021922	chr6p21.31	-	-	-1.31
*FANCF*	NM_022725	chr11p14.3	-	-	-1.72
*PALB2*	NM_024675	chr16p12.1	-	-	-1.32
*REV3L*	NM_002912	chr6q21	-	1.41	-
*HLTF*	NM_003071	chr3q25.1	-	-	-1.37
*PER1*	NM_002616	chr17p13.1	-	-	1.77
**B. Cell cycle and apoptosis**					
*GADD45B*	NM_015675	chr19p13.3	-	1.79	2.04
*DDIT3*	NM_004083	chr12q13.1	-	1.55	1.96
*BTG3*	NM_001130914	chr21q21.1	-	-	1.73
*PLK3*	NM_004073	chr1p34.1	-	-	1.79
*CDKN1A*	NM_000389	chr6p21.2	-	1.34	1.48
*BANP*	NM_001173539	chr16q24.2	-	1.31	1.49
*MDM4*	NM_002393	chr1q32	-	1.59	-
*MDM2*	NM_001145336	chr12q14.3	-	-1.48	-
*CCNG2*	NM_004354	chr4q21.1	-	-1.68	-1.69
*TP53BP1*	NM_001141979	chr15q15-q21	-	-	1.36
*ATM*	NM_000051	chr11q22-q23	-	-	-1.33
*ATMIN*	NM_015251	chr16q23.2	-	-	-1.30
*RIF1*	NM_001177663	chr2q23.3	-	-1.33	-1.41
*BCL2*	NM_000633	chr18q21.33	-	-	-1.48
*WAC*	NM_018604	chr10p12.1	-	1.56	1.54
*DUSP10*	NM_007207	chr1q41	-	1.67	2.01
*DUSP1*	NM_004417	chr5q34	-	1.79	1.99
*CKAP2*	NM_001098525	chr13q14	-	-	1.58
*GADD45G*	NM_006705	chr9q22.1	-	-	1.39
*PPP2R5C*	NM_001161725	chr14q32	-	-	1.47
*PMAIP1*	NM_021127	chr18q21.32	-	1.52	2.12
*TSC22D2*	NM_014779	chr3q25.1	-	1.72	2.14
*BTG1*	NM_001731	chr12q22	-	1.57	1.96
**C. Immune response**					
*KIR3DL1*	NM_006737	chr19q13.4	-	-	2.17
*IL1RAP*	NM_001167928	chr3q28	-	-	1.97
*IFNG*	NM_000619	chr12q14	-	-	2.46
*KIR3DS1*	NM_001083539	chr19q13.4	-	-	3.10
*KIR2DL5A*	NM_020535	chr19p13.3	-	-	2.05
*IL8*	NM_000584	chr4q13-q21	-	-	2.50
*IL1RN*	NM_000577	chr2q14.2	-	-	1.50
*IL16*	NM_001172128	chr15q26.3	-1.54	-1.66	-1.58
*IL5RA*	NM_000564	chr3p26-p24	-	-	-1.69
*BCL6*	NM_001130845	chr3q27	-	-	1.68
*TNFAIP6*	NM_007115	chr2q23.3	-	-	2.52
*TNFRSF10B*	NM_003842	chr8p22-p21	-	1.33	1.33
**D. Chromatin modification**					
*ACTR8*	NM_022899	chr3p21.1	-	-	-1.36
*ALKBH2*	NM_001001655	chr12q24.11	-	-	-1.35
*ASF1A*	NM_014034	chr6q22.31	-	-	-1.36
*UBE2B*	NM_003337	chr5q23-q31	-	1.34	1.36
*C16orf53*	NM_024516	chr16p11.2	-	-	-1.39
*RUVBL1*	NM_003707	chr3q21	-	-	-1.31
*ING5*	NM_032329	chr2q37.3	-	-	-1.49
*SETDB2*	NM_001160308	chr13q14	-	2.13	1.60
*MYST4*	NM_012330	chr10q22.2	-	1.40	-
*NCOA3*	NM_001174087	chr20q12	-	1.45	1.46
*JMJD6*	NM_001081461	chr17q25	-	-	1.75
*HIST1H1E*	NM_005321	chr6p21.3	-	1.85	-
*HIST1H2BC*	NM_003526	chr6p21.3	-	-	2.37
*HIST1H3A*	NM_003529	chr6p21.3	-	2.09	-
*KDM6B*	NM_001080424	chr17p13.1	-	1.33	2.00
*SIRT2*	NM_012237	chr19q13	-	-	1.90
*HDAC8*	NM_00116641	chrXq13	-	-	-1.31
*SUV420H1*	NM_016028	chr11q13.2	-	-1.61	-1.31
**E. Other important genes and transcription factors**					
*IER5*	NM_016545	chr1q25.3	-	-	1.55
*SOD2*	NM_000636	chr6q25.3	-	-	2.08
*JUN*	NM_002228	chr1p32-p31	-	1.93	2.30
*JUND*	NM_005354	chr19p13.2	-	1.7	1.93
*CREBZF*	NM_001039618	chr11q14	-	1.52	1.40
*ATF2*	NM_001880	chr2q32	-	1.38	1.39
*HEY1*	NM_001040708	chr8q21	-	-	1.53
*FOSB*	NM_001114171	chr19q13.32	-	-	2.38
*FOXO3*	NM_001455	chr6q21	-	1.33	1.42
*NR4A2*	NM_006186	chr2q22-q23	-	-	2.44
**B. Cell cycle and apoptosis**					
*GADD45B*	NM_015675	chr19p13.3	-	1.79	2.04
*DDIT3*	NM_004083	chr12q13.1	-	1.55	1.96
*BTG3*	NM_001130914	chr21q21.1	-	-	1.73
*PLK3*	NM_004073	chr1p34.1	-	-	1.79
*CDKN1A*	NM_000389	chr6p21.2	-	1.34	1.48
*BANP*	NM_001173539	chr16q24.2	-	1.31	1.49
*MDM4*	NM_002393	chr1q32	-	1.59	-
*MDM2*	NM_001145336	chr12q14.3	-	-1.48	-
*CCNG2*	NM_004354	chr4q21.1	-	-1.68	-1.69
*TP53BP1*	NM_001141979	chr15q15-q21	-	-	1.36
*ATM*	NM_000051	chr11q22-q23	-	-	-1.33
*ATMIN*	NM_015251	chr16q23.2	-	-	-1.30
*RIF1*	NM_001177663	chr2q23.3	-	-1.33	-1.41
*BCL2*	NM_000633	chr18q21.33	-	-	-1.48
*WAC*	NM_018604	chr10p12.1	-	1.56	1.54
*DUSP10*	NM_007207	chr1q41	-	1.67	2.01
*DUSP1*	NM_004417	chr5q34	-	1.79	1.99
*CKAP2*	NM_001098525	chr13q14	-	-	1.58
*GADD45G*	NM_006705	chr9q22.1	-	-	1.39
*PPP2R5C*	NM_001161725	chr14q32	-	-	1.47
*PMAIP1*	NM_021127	chr18q21.32	-	1.52	2.12
*TSC22D2*	NM_014779	chr3q25.1	-	1.72	2.14
*BTG1*	NM_001731	chr12q22	-	1.57	1.96
**C. Immune response**					
*KIR3DL1*	NM_006737	chr19q13.4	-	-	2.17
*IL1RAP*	NM_001167928	chr3q28	-	-	1.97
*IFNG*	NM_000619	chr12q14	-	-	2.46
*KIR3DS1*	NM_001083539	chr19q13.4	-	-	3.10
*KIR2DL5A*	NM_020535	chr19p13.3	-	-	2.05
*IL8*	NM_000584	chr4q13-q21	-	-	2.50
*IL1RN*	NM_000577	chr2q14.2	-	-	1.50
*IL16*	NM_001172128	chr15q26.3	-1.54	-1.66	-1.58
*IL5RA*	NM_000564	chr3p26-p24	-	-	-1.69
*BCL6*	NM_001130845	chr3q27	-	-	1.68
*TNFAIP6*	NM_007115	chr2q23.3	-	-	2.52
*TNFRSF10B*	NM_003842	chr8p22-p21	-	1.33	1.33
**D. Chromatin modification**					
*ACTR8*	NM_022899	chr3p21.1	-	-	-1.36
*ALKBH2*	NM_001001655	chr12q24.11	-	-	-1.35
*ASF1A*	NM_014034	chr6q22.31	-	-	-1.36
*UBE2B*	NM_003337	chr5q23-q31	-	1.34	1.36
*C16orf53*	NM_024516	chr16p11.2	-	-	-1.39
*RUVBL1*	NM_003707	chr3q21	-	-	-1.31
*ING5*	NM_032329	chr2q37.3	-	-	-1.49
*SETDB2*	NM_001160308	chr13q14	-	2.13	1.60
*MYST4*	NM_012330	chr10q22.2	-	1.40	-
*NCOA3*	NM_001174087	chr20q12	-	1.45	1.46
*JMJD6*	NM_001081461	chr17q25	-	-	1.75
*HIST1H1E*	NM_005321	chr6p21.3	-	1.85	-
*HIST1H2BC*	NM_003526	chr6p21.3	-	-	2.37
*HIST1H3A*	NM_003529	chr6p21.3	-	2.09	-
*KDM6B*	NM_001080424	chr17p13.1	-	1.33	2.00
*SIRT2*	NM_012237	chr19q13	-	-	1.90
*HDAC8*	NM_00116641	chrXq13	-	-	-1.31
*SUV420H1*	NM_016028	chr11q13.2	-	-1.61	-1.31
**E. Other important genes and transcription factors**					
*IER5*	NM_016545	chr1q25.3	-	-	1.55
*SOD2*	NM_000636	chr6q25.3	-	-	2.08
*JUN*	NM_002228	chr1p32-p31	-	1.93	2.30
*JUND*	NM_005354	chr19p13.2	-	1.7	1.93
*CREBZF*	NM_001039618	chr11q14	-	1.52	1.40
*ATF2*	NM_001880	chr2q32	-	1.38	1.39
*HEY1*	NM_001040708	chr8q21	-	-	1.53
*FOSB*	NM_001114171	chr19q13.32	-	-	2.38
*FOXO3*	NM_001455	chr6q21	-	1.33	1.42
*NR4A2*	NM_006186	chr2q22-q23	-	-	2.44

### Dose responsive genes in HLNRA individuals

**Fig 8:**
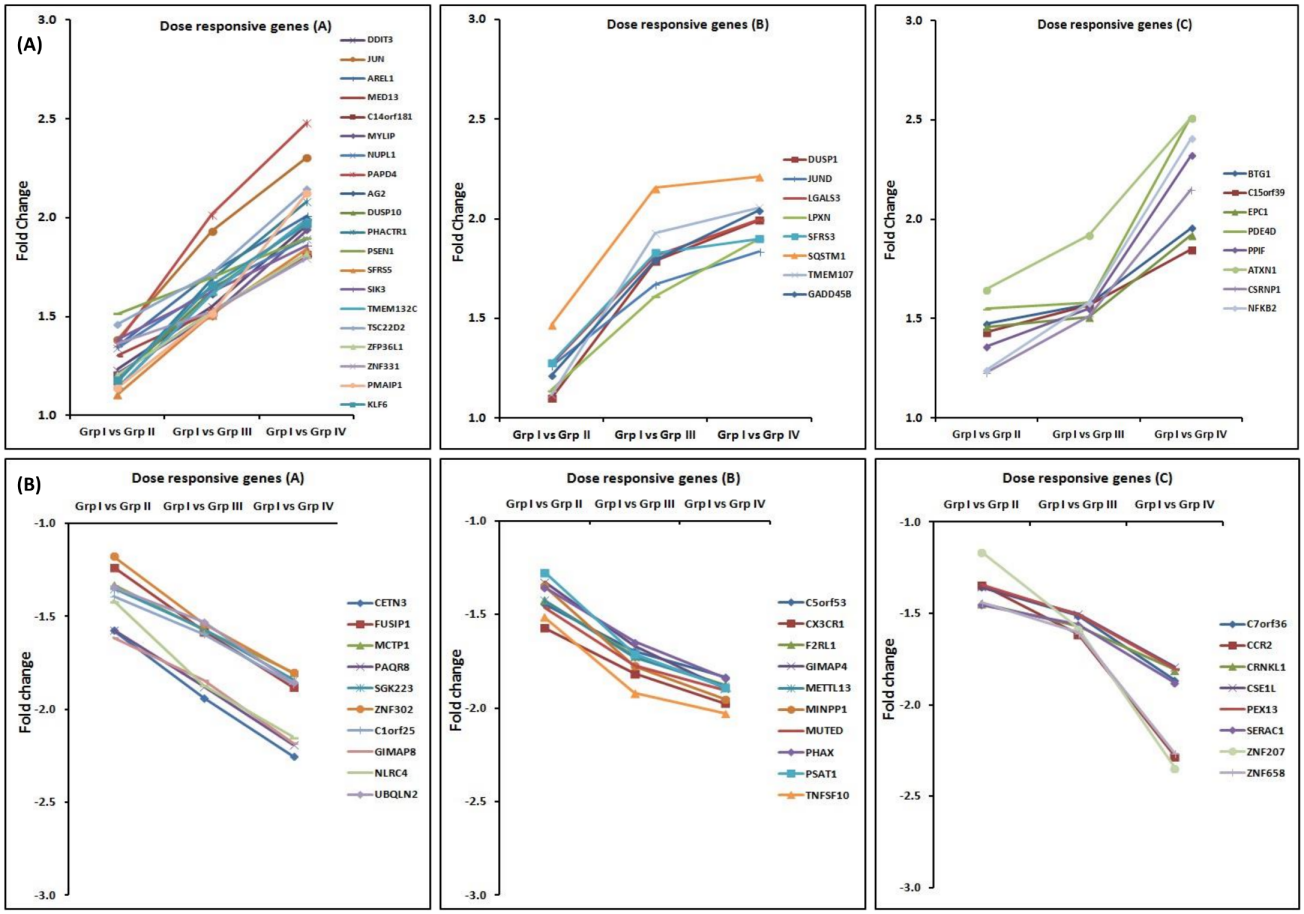
Dose responsive genes showing background dose dependent increase or decrease in expression among different dose groups. **(A)** Dose responsive genes showing background dose dependent increase in expression among different dose groups. (B) Dose responsive genes showing background dose dependent decrease in expression among different dose groups. Genes were divided in to three groups A, B and C on the basis of dose response trend shown by them. NLNRA (Group I), HLNRA (Group II, Group III and Group IV).

**Fig 9:**
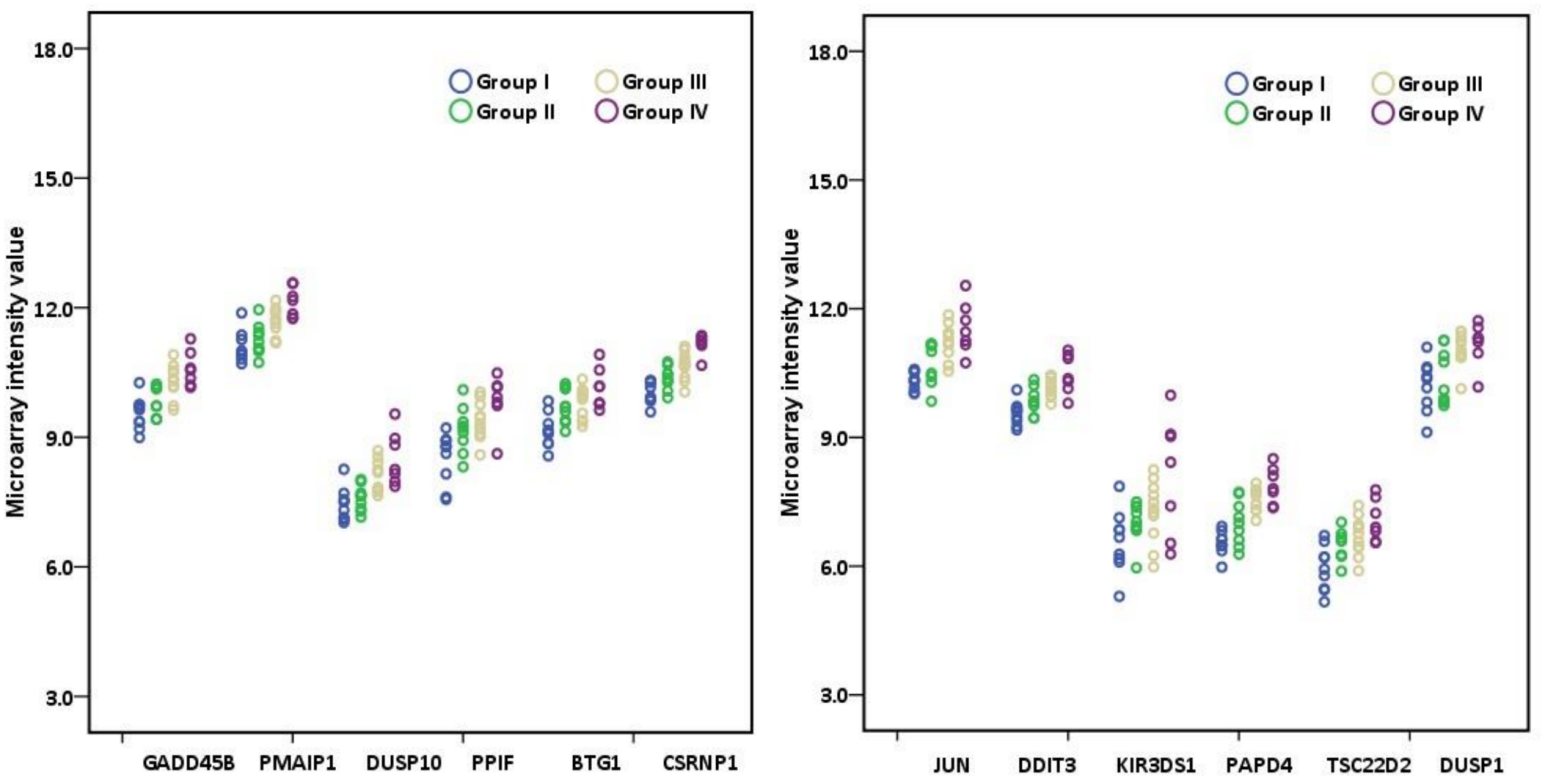
Scatter plot showing the expression level of different genes in the individuals belonging to different background dose groups (NLNRA, Group I and HLNRA. Group II, III and IV) in microarray analysis. Each dot represents normalized value in one individual. Different colored dots represent individuals belonging to different dose groups. Group I (N = 9), Group II (N = 9), Group III (N = 11), Group IV (N = 7).

We have also identified 64 genes (36 up-regulated and 28 down-regulated) which were highly expressed (≥ 2.0 fold) in Group IV and showed background radiation dose dependent increase or decrease (up and down regulation) in their expression profile. Fig 8 represents the fold changes in the expression of these genes in different background dose groups (Group I vs Group II, Group I vs Group III and Group I vs Group IV). Gene ontology analysis have revealed that most of these genes are involved in Cell cycle regulation *(GADD45B*, *BTG1*, *GNAS*, *GIMAP8*, *GIMAP4)*, immune system (*NFKB2*, *CXCR1*, *TNFSF10*, *CCR2*), stimulus to DNA damage *(DDIT3*, *DUSP1*, *JUN*, *GADD45B*, *JUND*, *BTG1)*, apoptosis *(PMAIP1*, *PPIF*, *TSC22D2*, *SIK3*, *NLRC4)*, mRNA processing *(CRNKL1*, *SFRS3*, *SFRS5*, *FUSIP1*, *PHAX)*, transcriptional regulation *(JUN*, *JUND*, *KLF6*, *ATXN1*, *MED13*, *MED26*, *ZNF302*, *NFKB2 ZNF207*, *ZNF658)* and signal transduction *(DUSP1*, *DUSP10*, *LPXN*, *MYLIP*, *LGALS3*, *PAQR8)* and ubiquitin dependent proteolysis *(SQSTM1*, *UBQLN2)* etc. A representative scatter plot showing the microarray intensity values of selected genes in individuals of different background dose groups is given in Fig 9.

### Validation of differentially expressed genes from microarray data using RT q-PCR

Thirty differentially expressed genes (22 up-regulated and 8 down-regulated) were validated using RT q-PCR. Validation of these genes was carried out in 54 individuals, which include 30 individuals used for microarray analysis and another independent set of 24 individuals from the same dose groups of Kerala population. These genes were selected from different dose group comparisons involved in various biological processes, having different expression levels in order to get a good representation from microarray data. These include *PMAIP1*, *PAPD4*, *DDIT3*, *GIMAP8*, *PPIF*, *CSRNP1*, *BTG1*, *CDKN1A*, *DUSP10*, *GADD45B*, *TSC22D2*, *METTL13*, *cJUN*, *DUSP1*, *KIR3DS1*, *JUND*, *EIF1*, *ATXN1*, *SNRPA1*, *DHFRL1*, *NAMPT*, *ZNF167*, *THAP2*, *KLF6*, *BBS10*, *PLK3*, *PDK4*, *SETDB2*, *KDM6B*, *CCR2*.

**Fig 10:**
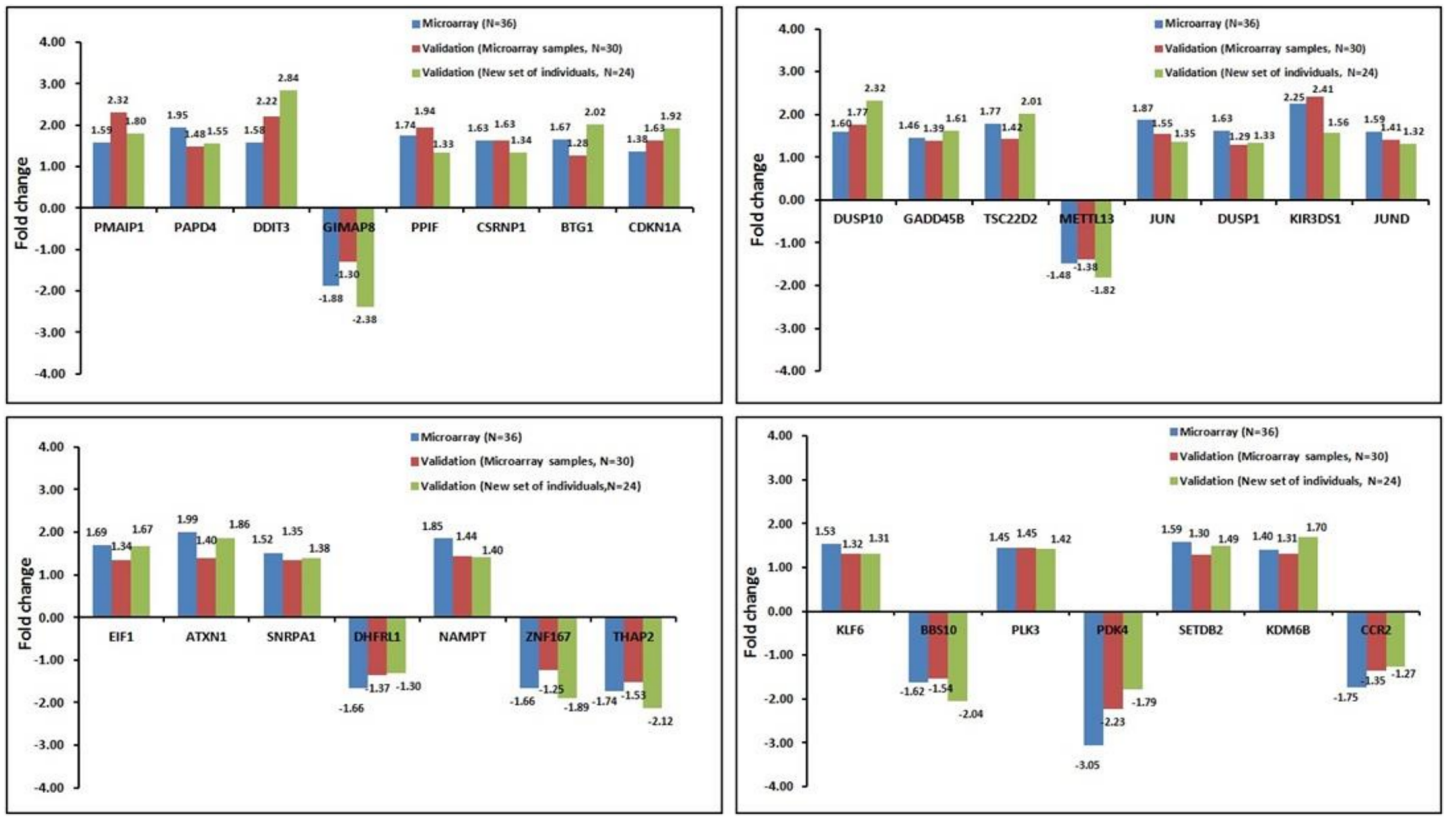
Validation of differentially expressed genes using RT q-PCR experiment. Comparison of fold change values obtained in two different sets of individuals along with microarray results are shown. Blue bar represents microarray results, Red bar shows RT q-PCR results of microarray samples, Green bar shows RT q-PCR results of new set of samples. Fold change values are shown above each bar in all the gene.

As shown in Fig 10, the average fold change values obtained from microarray experiments from 36 individuals. The fold change values obtained from RT q-PCR experiments in 30 individuals (microarray samples) and 24 individuals (new set of individuals) showed similar trend and good correlation for all the 30 genes studied. Our results showed consistent gene expression profile of the genes in microarray experiment as well as RT q-PCR experiments. We also observed similar expression values of the genes in new random set of individuals suggesting the results obtained are consistent in the population.

## Discussion

The present study is focused on the cellular responses to chronic low dose and low dose-rate radiation in humans. The baseline gene expression is very important to understand the *ex-vivo/ in-vivo* effect of low dose and dose-rate exposure ^55^. Human population living in high level natural radiation areas (HLNRA) is exposed to chronic low dose background radiation since generations and provides unique opportunity to understand the *in-vivo* effects of low dose radiation exposure directly on humans. Most importantly, the unique feature of HLNRA of Kerala coast is the non-uniform distribution of monazite in the beach sand which leads to varying level of background radiation (<1.0 to 45mGy/year) along the 55 km long stretch allowing us to investigate *in-vivo* dose response, if any at various biological end points. Several epidemiological (congenital malformation, cancer incidence etc) and biological studies carried out in this population have not revealed any significant difference between NLNRA and HLNRA population [9,11,16,17,18,20,22]. However, recent studies have shown a lower induction and efficient repair of DNA damage in HLNRA individuals [21,23].

In the present study, global gene expression profile was carried out to understand biological response of human cells exposed to chronic low dose and low dose-rate radiation. The individuals included in the study are classified into four different background dose groups based on the individual dose received annually by them. The first group receives a dose ≤ 1.5mGy/year and are considered as control population (NLNRA) and the other three groups are from HLNRA receiving a dose >1.5mGy/year. Our results have shown that a large number of genes are differentially expressed in HLNRA individuals as compared to NLNRA. Interestingly, a dose dependent increase in the number of differentially expressed genes with respect to background radiation levels was observed in HLNRA groups. The plausible explanation could be that individuals belonging to higher background dose groups (> 5.0mGy/year) have accumulated larger doses and thus stimulating many genes from different cellular and molecular pathways to maintain genome integrity. Further, detailed gene ontology analysis revealed an over-representation of genes involved in DNA damage response (DDR), DNA repair, cell cycle regulation, mRNA processing, protein transport, stress response, histone/chromatin modification, methylation, apoptosis, transcriptional regulation, signal transduction, and immune response in individuals belonging to higher dose groups (Group III and Group IV) of HLNRA. The above findings are supportive of the results obtained from DNA damage and repair study in HLNRA, where significantly lower induction of DNA damage and efficient repair of DNA strand breaks observed in individuals belonging to higher dose groups (>5.0 mGy/year) of HLNRA [18,21,23].

Our results have also shown that, Group II (≤ 5.0mGy/year) has very few significantly differentially expressed genes as compared to Group III and Group IV of HLNRA (>5.0mGy/year). Most of these genes are involved in transcriptional regulation, protein transport and RNA processing. Interestingly, an abundance or over-representation of DDR and DNA repair genes was observed in higher dose groups (>5.0 mGy/year). These included DNA repair genes such as *UNG* and *APEX2* involved in base excision repair, *RAD23B* and *ERCC4* genes which play important role in nucleotide excision repair, PMS1 from mismatch repair pathway and *DCLRE1C* (Artemis), *XRCC4*, *LIG4* genes involved in NHEJ pathway. The differentially expressed genes clearly indicate the active involvement of all the molecular pathways of DNA repair in HLNRA individuals (>5.0 mGy/year). For instance, *APEX2* is the regulatory gene of BER which controls both short patch and long patch pathways. Uracil DNA glycosylase (*UNG*) is the major glycosylase for the removal of uracil from DNA and initiates base excision repair [58] indicating that BER is active in HLNRA population. Similarly, presence of *DCLRE1C* (Artemis), *XRCC4*, *LIG4* indicates that DSB repair is occurring in HLNRA and perhaps mediated through artemis-dependent pathway. Additionally, differential expression of *ERCC4* and *RAD23B* suggests possible involvement of nucleotide excision repair pathway. Activation of genes involved in DDR response (*DDIT3*, *SOD2*), cell cycle check points (*GADD45*, *CDKN1A*, *BTG1*, *BTG3*, *CCNG2*), apoptosis (*PMAIP1*, *PPP1R15A*, *PPP2R5C*, *SIAH1*, *APAF1*) and signal transduction pathways such as mitogen activated protein kinase (MAPK) pathway, Jun amino-terminal kinases (JNK) pathway, p53 pathway, T cell receptor signaling, B-cell receptor signaling, Jak-stat signaling, purine metabolism, ubiquitin mediated proteolysis, and gap junction etc are suggestive of DDR to be active in this population exposed to chronic low dose radiation.

Our observation that more number of DNA damage or stress response genes in higher background dose groups suggests that perhaps a certain level of minimum damage is required for the cells to sense and activate DDR signaling [4,59]. Our results indicate a threshold dose of ~ 5.0mGy/year for chronic low dose exposure in HLNRA population for activation of molecular pathways of DNA damage response and better cell survival.

The signal transduction pathways such as mitogen activated protein kinase (MAPK) pathway, Jun amino-terminal kinases (JNK) pathway and p53 pathway have been shown to play an important role in low dose radiation induced adaptive response [60,61,62]. Our results have shown over-representation of genes involved in MAPK pathway (*DUSP1*, *DUSP10*, *AKT2*, *ATF2*, *DDIT3*, *DUSP5*, *GNAS*, *HIPK3*, *IL8* etc.) and p53 pathway (*CDKN1A*, *MDM2*, *TRAF4*, *TNFRSF10B*, *APAF1*, *PMAIP1*, *GADD45B* etc.) in higher dose groups of HLNRA (>5.0 mGy/year). Several known radiation responsive genes such as *CDKN1A* and *GADD45B* etc involved in cell cycle check point activation were upregulated in HLNRA individuals.

Immune response, cell to cell communication and gap junction genes and proteins are known to play important role in radio-adaptive response [63,64,65]. However, such information on PBMC at G_0_/G_1_ exposed to chronic low dose exposure is limited. In the present study, representation of genes involved in modulation of immune response (*TNFRSF10B*, *IL16*, *IL8*,*CCR2*, *KIR3DS1*, *IFNG* etc.), cell-cell communication and gap junction (*TUBB3*, *GNAS*, *TUBB2C*, *LPAR1* etc) in HLNRA population suggest their possible role in radio-adaptive response.

Another interesting finding is the transcriptional induction of a number of transcription factors such as *c-JUN*, *JUND*, *FOS*, *ATF2*, *NR4A2*, *Sp1*, *FOXO3*, *CREBZF*, which are known to play important role in DDR signaling and repair. Genes such as c*JUN*, *JUND*, *FOS*, *ATF2*, *ATF3* and *CREBZF* belong to activating protein 1 (AP1) family of transcription factors (TFs). AP1 family TFs are stress responsive transcription activators and has been implicated in DNA repair by its ability to regulate a large set of genes functioning in DNA repair [66]. Recently, it has been shown that TFs such as ATF2 and NR4A2 not only regulates the transcription of genes involved in DNA repair, but also translocate to the site of DNA lesion and play a direct role in DNA repair [66,67]. NR4A2 has been shown to get translocated to DNA repair foci, where it gets phosphorylated by DNA-PKcs and co-localizes with other repair proteins such as gamma-H2AX, DDB2 and XPC [68,69] Increased expression of these transcription factors in higher dose groups of HLNRA individuals are suggestive of active involvement of DDR signaling and repair in high dose groups of HLNRA individuals (> 5.0 mGy/year).

Studies on alteration of gene expression profile in human cells in response to low dose ionizing radiation are limited. Few studies have been carried out where transcriptional response in occupational workers exposed to very low dose ionizing radiation have been reported [41,50]. Morandi et al (2009) reported over-representation of histone modification genes involved in DNA packaging, chromatin architecture and DNA metabolism in medical workers exposed to low doses of radiations[50]. The role of histone modification/chromatin changes in DNA repair after acute exposure to IR has been reported [70,71,72,73]. In our study, we also observed large number of genes (*HIST1H1E*, *H3F3B*, *HIST1H2BC*, *HP1BP3*, *KDM6B*, *ING3*, *MYST4*, *SETDB2*, *MECP2*, *MLL*, *SUV420H1 etc)* involved in epigenetic processes such as histone/chromatin modification, DNA packaging, methylation, and DNA metabolism to be over-represented. It indicates the alteration of chromatin structure is one of the important cellular response to chronic low dose exposure. Further, Fachin et al. (2009) observed several biological processes such as ubiquitin cycle (*UHRF2* and *PIAS1*), DNA repair (*LIG3*, *XPA*, *ERCC5*, *RAD52*, *DCLRE1C*), cell cycle regulation/proliferation (*RHOA*, *CABLES2*, *TGFB2*, *IL16*), and stress response (*GSTP1*, *PPP2R5A*, *DUSP22*) to be active chronic low level radiation exposures[41]. Interestingly, such pathways are over-represented in our data suggesting their response to chronic low dose radiation exposure.

We have also identified a set of dose responsive genes *DDIT3*, *GADD45B*, *JUN*, *PMAIP1*, *DUSP1*, *PAPD4*, *DUSP10*, *BTG1*, *PPIF*, *TSC22D2*, *TMEM132C*, *GIMAP8*, *METTL13*, *CSRNP1*, *KIR3DS1*, *TNFSF10* etc. which have potential to be used as possible low dose radiation signatures in humans.

In summary, our findings clearly indicated that individuals exposed to background doses of >5mGy/year have shown alteration in expression of many genes involved in important functions or pathways. These included DDR signaling, DNA repair, RNA metabolism, epigenetic changes (histone/chromatin modifications), cell cycle regulation, immune response, apoptosis etc. These may be the primary reason of not getting any detectable change at phenotype or DNA damage levels in HLNRA individuals exposed to chronic low level background dose exposures.

Sixty four dose responsive genes were identified, which are the possible radiation signatures for chronic low dose radiation exposure. In conclusion, global gene expression profile in response to natural chronic low dose radiation revealed active DDR and repair processes and their interaction with various cellular and molecular pathways in HLNRA individuals belonging to higher dose groups >5mGy/year. These findings indicated a possible threshold dose of 5mGy/year for signaling DDR and a plausible reason of observing *in vivo* radio-adaptive response and non-carcinogenesis in HLNRA population. These findings have tremendous implications in understanding the molecular effect of low dose radiation biology, especially the effect of low dose radiation in humans at low dose region.

## Supporting information

S1 Table Details of primer sequences and UPL probe numbers of the genes studied using real time PCR. (DOCX)
