# Supplementary material for: Global transcriptome profile reveals abundance of DNA damage response and repair genes in individuals from high level natural radiation areas of Kerala coast
Source: PLoS One. 2017 Nov 21;12(11):e0187274. doi: 10.1371/journal.pone.0187274 (PMC5697823; doi:10.1371/journal.pone.0187274)
Supplement: S1 Table — (DOCX) [file pone.0187274.s001.docx]

|  | | | |
| --- | --- | --- | --- |
| S1 Table: ~~Supplementary table 1:~~  Details of primer sequences and UPL probe numbers of the genes studied using Real time PCR. | | | |
| Gene  Symbol | Primer Sequence ( 5’ - 3’) | Base pair  (bp) | UPL probe no. |
| *SNRPA1* | PR1 5’ tcc gca agt cag agt act gg 3’  PR2 5’ ccg ttt gcc ctt gaa cat t 3’ | 20  19 | 38 |
| *METTL13* | PR1 5’ GCT GAG CTG TCG GCT AGA GT 3’  PR2 5’ ACA GAC AGA AAG GGG ACC TG 3’ | 20  20 | 33 |
| *ATXN1* | PR1 5’ GAG CCT GTG GGA AGTCTC C 3’  PR2 5’ CAA CAG CAG CTC TGG ATG AA 3’ | 19  20 | 30 |
| *NAMPT* | PR1 5’ gcc agc agg gaa ttt tgt ta 3’  PR2 5’ gcc att ctt gaa gac agt atg ga 3’ | 20  23 | 65 |
| *KIR3DS1* | PR1 5’ ggt caa aat ccc ttt cac ca 3’  PR2 5’ tgg tcc att aca gca gca tt 3’ | 20  20 | 35 |
| *DHFRL1* | PR1 5’ tga tgc ctt aaa act tac tga acg 3’  pr2 5’ atg gcc tag gtg att cat gg 3’ | 24  20 | 87 |
| *PDK4* | PR1 5’ cag tgc aat tgg tta aaa gct g 3’  PR2 5’ tgg tca tct ggg ctt ttc tc 3’ | 22  20 | 31 |
| *GADD45B* | PR1 5’ cat tgt ctc ctg gtc acg aa 3’  PR2 5’ tag ggg acc cac tgg ttg t 3’ | 20  19 | 10 |
| *CDKN1A* | PR1 5’ cga agt cag ttc ctt gtg gag 3’  PR2 5’ cat ggg ttc tga cgg aca t 3’ | 21  19 | 82 |
| *PLK3* | PR1 5’ gaa ggt ggg gga ttt tgg 3’  PR2 5’ ggg tgc cac aga tgg tct 3’ | 18  18 | 6 |
| *DDIT3* | PR1 5’ cag agc tgg aac ctg agg ag 3’  PR2 5’ tgg atc agt ctg gaa aag ca 3’ | 20  20 | 9 |
| *JUND* | PR1 5’ cag cga gga gca gga gtt 3’  PR2 5’ gag ctg gtt ctg ctt gtg taa at 3’ | 18  23 | 81 |
| *KLF6* | PR1 5’ ctt ctc aac tgt ggg gtt gc 3’  PR2 5’ ttc cca tga gca tct gta agg 3’ | 20  21 | 2 |
| *EIF1* | PR1 5’cca gaa cct cca ctc ttt cg 3’  PR2 5’gcc gtt tct ctg ttg aat tct t 3’ | 20  22 | 04 |
| *ZNF167* | PR1 5’ tgg aaa agt ctc aca ctc agt ca 3’  PR2 5’ ccc ttc atc atg ttc tca cct 3’ | 23  21 | 86 |
| *BTG1* | PR1 5’tgc aga cct tca gcc aga g 3’  PR2 5’ATC CCT tgc atg gct ttt c 3’ | 19  19 | 17 |
| *THAP2* | PR1 5’ttg acc taa cag gac aaa ctc g 3’  PR2 5’aaa aga ttc ctt gac ttg agt ttc a 3’ | 22  25 | 89 |
| *KDM6B* | PR1 5’cac cca ctg tgg tct gtt gt 3’  PR2 5’tgt ctc cgc ctc agt aac ag 3’ | 20  20 | 3 |
| *PAPD4* | PR1 5’tgg aaa tgt cag tcc aat aca ga 3’  PR2 5’acc gtc aag agg aag gtt ttt 3’ | 23  21 | 60 |
| *PMAIP1* | PR1 5’gga gat gcc tgg gaa gaa g 3’  PR2 5’cct gag ttg agt agc aca ctc g 3’ | 19  22 | 67 |
| *TSC22D2* | PR1 5’tga aca gtc tgg cca cct c 3’  PR2 5’acg ctt ggt aga aag cag ttg 3’ | 19  21 | 9 |
| *DUSP10* | PR1 5’tga atg tgc gag tcc ata gc 3’  PR2 5’tgg caa ttc aag aag aac tca a3’ | 20  22 | 22 |
| *GIMAP8* | PR1 5’acg gag gac cct atc atg tg 3’  PR2 5’tgc agc ttc att cac aca atc 3’ | 20  21 | 8 |
| JUN | PR1 5’cca aag gat agt gcg atg ttt3’  PR2 5’ctg tcc ctc tcc act gca ac3’ | 21  20 | 19 |
| *DUSP1* | PR1 5’ttc aac gag gcc att gac tt 3’  PR2 5’cct ggc agt gga caa aca c 3’ | 20  19 | 65 |
| *CSRNP1* | PR1 5’cct gcc tga ccg tga ctt 3’  PR2 5’agc ccg ctt cag gat aga c 3’ | 18  19 | 57 |
| *PPIF* | PR1 5’ gaa ggg ctt cgg cta caa a 3’  PR2 5’ cat tgt ggt tgg tga agt cg 3’ | 19  20 | 39 |
| *CCR2* | PR1 5’ tga gac aag cca caa gct ga 3’  PR2 5’ ttc tga taa acc gag aac gag at 3’ | 20  23 | 56 |
| *BBS10* | PR1 5’ ttc tca gcc gga atg gag 3’  PR2 5’ gat gac tgg aaa cac agt cca c 3’ | 18  22 | 79 |
| *SETDB2* | PR1 5’ggc att ctt cac caa cag gt 3’  PR2 5’ccc aca ttg gca gaa gat tt 3’ | 20  20 | 33 |
| *β -ACTIN* | PR1 5’cca acc gcg aga aga tga 3’  PR2 5’cca gag gcg tac agg gat ag 3’ | 18  22 | 64 |
